# Pre-Treatment Objective Diagnosis and Post-Treatment Outcome Evaluation in Patients with Vascular Pulsatile Tinnitus Using Transcanal Recording and Spectro-Temporal Analysis

**DOI:** 10.1371/journal.pone.0157722

**Published:** 2016-06-28

**Authors:** Shin Hye Kim, Gwang Seok An, Inyong Choi, Ja-Won Koo, Kyogu Lee, Jae-Jin Song

**Affiliations:** 1 Department of Otorhinolaryngology-Head and Neck Surgery, Seoul National University Bundang Hospital, Seongnam, Korea; 2 Department of Otorhinolaryngology-Head and Neck Surgery, Korea University Medical Center, Seoul, Korea; 3 Music and Audio Research Group, Graduate School of Convergence Science and Technology, Seoul National University, Seoul, Korea; 4 Department of Communication Sciences and Disorders, University of Iowa, Iowa City, IA, United States of America; University of Regensburg, GERMANY

## Abstract

**Objective:**

Although vascular pulsatile tinnitus (VPT) has been classified as “objective”, VPT is not easily recognizable or documentable in most cases. In response to this, we have developed transcanal sound recording (TSR) and spectro-temporal analysis (STA) for the objective diagnosis of VPT. By refining our initial method, we were able to apply TSR/STA to post-treatment outcome evaluation, as well as pre-treatment objective diagnosis.

**Methods:**

TSR was performed on seven VPT patients and five normal controls before and after surgical or interventional treatment. VPT was recorded using an inserted microphone with the subjects placed in both upright and supine positions with 1) a neutral head position, 2) head rotated to the tinnitus side, 3) head rotated to the non-tinnitus side, and 4) a neutral position with ipsi-lesional manual cervical compression. The recorded signals were analyzed in both time and time-frequency domains by performing a short-time Fourier transformation.

**Results:**

The pre-treatment ear canal signals of all VPT patients demonstrated pulse-synchronous periodic structures and acoustic characteristics that were representative of their presumptive vascular pathologies, whereas those the controls exhibited smaller peaks and weak periodicities. Compared with the pre-treatment signals, the post-treatment signals exhibited significantly reduced peak- and root mean square amplitudes upon time domain analysis. Additionally, further sub-band analysis confirmed that the pulse-synchronous signal of all subjects was not identifiable after treatment and, in particular, that the signal decrement was statistically significant at low frequencies. Moreover, the post-treatment signals of the VPT subjects revealed no significant differences when compared to those of the control group.

**Conclusion:**

We reconfirmed that the TSR/STA method is an effective modality to objectify VPT. In addition, the potential role of the TSR/STA method in the objective evaluation of treatment outcomes in patients with VPT was proven. Further studies incorporating a larger sample size and more refined recording techniques are warranted.

## Introduction

Tinnitus can be classified as either non-pulsatile subjective or pulsatile. Non-pulsatile subjective tinnitus originates from the inner ear, ascending auditory pathway, or cortical regions [1–6], whereas pulsatile tinnitus (PT) is defined as tinnitus with heartbeat-synchronous and periodic nature [7,8]. The causes of PT may be divided into vascular and nonvascular causes. Vascular pulsatile tinnitus (VPT) is produced from turbulent blood flow, which is transmitted directly to the inner ear [9], whereas nonvascular PT arises from abnormal muscle contraction [10].

PT is frequently equated with “objective tinnitus”, since some cases of PT can be detected by an observer [7,8]. However, considering that only 20% of all PT cases are objectively detected by clinicians [11], most cases of PT remain subjective. As a result, the pre-treatment evaluation of PT and the post-treatment assessment of outcomes usually rely on the subjective accounts of patients. Additionally, the initial choice of imaging modality, such as temporal bone computed tomography angiography (CTA), brain magnetic resonance imaging/angiography (MRI/A), or trans-femoral cerebral angiography (TFCA), is also based on otoscopic findings or subjectively-perceived changes in loudness by head rotation, cervical compression, or the Valsalva maneuver [12–14]. Thus, when a patient complains of PT but the PT is inaudible by auscultation and the imaging findings are equivocal, surgeons may encounter difficulty in choosing the appropriate management options.

The aim of precise evaluation for patients with VPT is to demonstrate treatable causes, since most causes of VPT are curable with precise evaluation and proper management of the responsible vascular structure [15]. We recently developed a novel method of transcanal sound recording (TSR) and spectro-temporal analysis (STA) for the objective and differential diagnosis of VPT. Our initial study [16] demonstrated that the TSR/STA method may provide additional information regarding the origins of particular cases of VPT, as well as being an efficient and objective diagnostic tool. However, that study lacked a pre- and post-treatment comparison of the recorded sound. In addition, we also refined the recording unit in a number of ways. Therefore, the current study aims to implement the refined TSR/STA method not only for pre-treatment objective diagnosis, but also for the post-treatment evaluation of changes in patients with VPT due to various vascular pathologies.

## Materials and Methods

### Participants

This study comprised seven unilateral VPT patients who underwent surgical treatment at Seoul National University Bundang Hospital between January 2015 and August 2015 and five volunteered control subjects with no complaints of VPT. Patients were asked to note the tinnitus handicap inventory (THI) score [17], numeric rating scale (NRS) loudness (answering to a question “how loud is your tinnitus?” on a scale from 0 to 10), NRS distress (answering to a question “how bothered are you by your tinnitus?” on a scale from 0 to 10) character, and factors that increased or decreased the loudness of their VPT. All patients underwent otoendoscopic examination and audiologic tests, including pure tone audiometry, speech audiometry, tympanometry, and stapedial reflex. Additionally, temporal bone CTA, brain MRI/A, and/or TFCA were performed to identify possible vascular structural abnormalities relevant to the patient’s perception of VPT. To evaluate the post-treatment changes, the same questionnaires (THI score, NRS loudness, and NRS distress), audiologic tests, and/or imaging examination were again performed 3 months after surgical treatment. The study was approved by the institutional review board of the Clinical Research Institute at Seoul National Bundang Hospital (B-1601-329-002) and all participants provided their written informed consent.

### Sound recording

TSR was performed on all VPT subjects and controls. For the VPT subjects, the measurements were performed before and 3 months after surgical treatment. Pre-treatment recordings were performed before imaging examinations such as temporal bone CTA, brain MRI/A, and/or TFCA. VPT was measured in eight different head positions: 1) a neutral head position, 2) head rotated to the tinnitus side, 3) head rotated to the non-tinnitus side, and 4) a neutral position with manual cervical compression of the tinnitus side, all the four in both upright and supine positions. For the controls, the measurements were performed in the same positions as for the VPT subjects. To record the sound pressure waves generated by VPT, an external auditory canal (EAC)-sealing microphone was developed. We chose a type of small diaphragm condenser microphone (LAVALIER, 12.00 mmH x 4.50 mmW x 4.50 mmD, RODE, Sydney, Australia) that was small enough to fit into the EAC. Additionally, an in-ear foam tip was attached to the end of the microphone to prevent noise input through the complete sealing of the subject’s EAC.

The microphone has flat frequency responses up to 18 kHz with a sensitivity of -33.5 dB re 1 V/Pa (21.00 mV at 94 dB sound pressure level (SPL)), according to the accompanying datasheet. The signals were recorded using a battery-operated portable digital recorder (H4N, ZOOM, Tokyo, Japan) at a sampling rate of 44,100 Hz and the recorded signals were analyzed using MATLAB R2013a (MathWorks, Natick, MA, USA). To achieve a highly selective recording system, a battery-powered recording device was required to prevent noise generated from electricity or a magnetic field that may cause a ground loop problem.

### Temporal analysis (time domain analysis)

The acquired signals were filtered by a Butterworth high-pass filter with a cut-off frequency of 20 Hz. The peak amplitudes were computed by finding the maximum instantaneous absolute value from the filtered signals, and a root mean square (RMS) amplitude was used to represent the effective output of the signal. To quantify the differences between pre- and post-treatment ear canal signals, the peak amplitudes and RMS amplitudes were calculated using 5-second-long periods. The Wilcoxon signed-rank sum test was used to analyze the differences between pre- and post-treatment ear canal signals with regard to peak amplitudes and RMS amplitudes. Statistical significance was set at a *P*-value < 0.05.

### Spectro-temporal analysis (time-frequency domain analysis)

STA was performed with a short-time Fourier transform at a frequency range of 20 to 8,000 Hz. A Hanning window of ~50 ms (2,048 samples) was used with a hop size of ~10 ms (512 samples). As the sounds were sampled by the digital recorder, the magnitudes of the frequencies were converted to dB SPL using a microphone calibrator. The frequency range was divided into 1/2-octave bands and five pulse-synchronized spectral bands (125, 250, 500, 1,000, and 2,000 Hz) were chosen for the statistical observations for the comparison of changes in signal intensities between pre- and post-treatment signals. The Wilcoxon signed-rank sum test was used to analyze SPL differences in the five pulse-synchronized spectral bands between the pre- and post-treatment signals, whereas the Mann Whitney U-test was used to evaluate the differences between the pre-treatment signals and the control subjects’ signals and between the post-treatment signals and the control subjects’ signals. A *P*-value < 0.05 was considered to indicate statistical significance.

## Results

### Demographic characteristics and presumed underlying pathology of the subjects

The demographic characteristics of the seven VPT patients are summarized in Table 1. Of the seven subjects, four were female and one was male, with a median age of 52 years (range, 23–63 years). All seven subjects complained of right-sided PT. On otoendoscopic examination, all subjects displayed normal tympanic membrane and external ear canal findings. Pure tone audiometry indicated a normal range for the hearing threshold (< 20 dB HL for 250 to 8,000 Hz) in six subjects, while Subject 5 showed decreased ipsi-lesional low frequency thresholds (30dB at 250 and 500 Hz).

**Table 1:** Demographic characteristics of the included subjects with vascular pulsatile tinnitus.

No.	Sex/ Age	Onset	Side	Conditions increasing the VPT loudness	Conditions decreasing the VPT loudness	Diagnosis	Surgical treatment	Changes in the THI score after treatment	Changes in the NRS loudness after treatment	Changes in the NRS distress after treatment
1	F/53	5YA	Rt		Rt. Neck compression	Sigmoid sinus diverticulum	Sigmoid sinus resurfacing	From 78 to 0	From 8 to 0	From 8 to 0
2	F/52	6MA	Rt		Head rotation to rt.	Dehiscent jugular bulb	Jugular bulb resurfacing	From 70 to 4	From 9 to 2	From 10 to 0
3	F/40	3MA	Rt		When administration of medicine for blood circulation	Dominant transverse-sigmoid sinus with high jugular bulb	Sigmoid sinus reshaping	From 50 to 44	From 9 to 2	From 9 to 5
4	M/40	6MA	Rt	Bowing		Dural arteriovenous fistula	Transarterial embolization	From 50 to 0	From 7 to 0	From 7 to 0
5	F/63	1YA	Rt	Head rotation to lt.	Rt. Neck compression,head rotation to rt.	Sigmoid sinus diverticulum	Sigmoid sinus resurfacing	From 68 to 0	From 8 to 0	From 8 to 0
6	F/43	6MA	Rt	Head rotation to lt.	Rt. Neck compression,head rotation to rt.	Dehiscent jugular bulb	Jugular bulb resurfacing	From 30 to 26	From 5 to 2	From 7 to 3
7	F/23	6MA	Rt	Head rotation to lt.	Rt. Neck compression,head rotation to rt.	Dominant transverse-sigmoid sinus	Sigmoid sinus reshaping	From 46 to 0	From 7 to 0	From 6 to 0

No., number; F, female; M, male; YA, years ago; MA, months ago; Rt., right; Lt., left; THI, tinnitus handicap inventory; NRS, numeric rating scale.

Temporal bone CTA and/or brain MRI/A revealed a right sigmoid sinus diverticulum in Subjects 1 and 5, a dehiscent high jugular bulb in Subject 2 and 6, a dominant transverse sigmoid sinus without bony dehiscence with high jugular bulb in Subject 3, a dominant transverse sigmoid sinus with bony dehiscence in Subject 7, and a dural arteriovenous fistula in Subject 4 (Table 1). According to their vascular structure abnormalities, Subjects 1 and 5 underwent resurfacing of the sigmoid sinus as described in our previous report [14], Subject 2 and 6 resurfacing of the jugular bulb via transcanal approach using bone cement, Subject 3 and 7 reshaping of the sigmoid sinus as described in our previous report [13], and Subject 4 transarterial embolization of the dural arteriovenous fistula. Immediately after surgical treatment, VPT completely disappeared in four of the seven subjects (Subjects 1, 4, 5, and 7) or was much abated (Subjects 2, 3 and 6). Moreover, the improvement in symptoms persisted 3 months after surgical treatment in all patients. The changes in the THI score, NRS loudness, and NRS distress are summarized in Table 1.

### Temporal analysis (time domain analysis)

**Fig 1:**
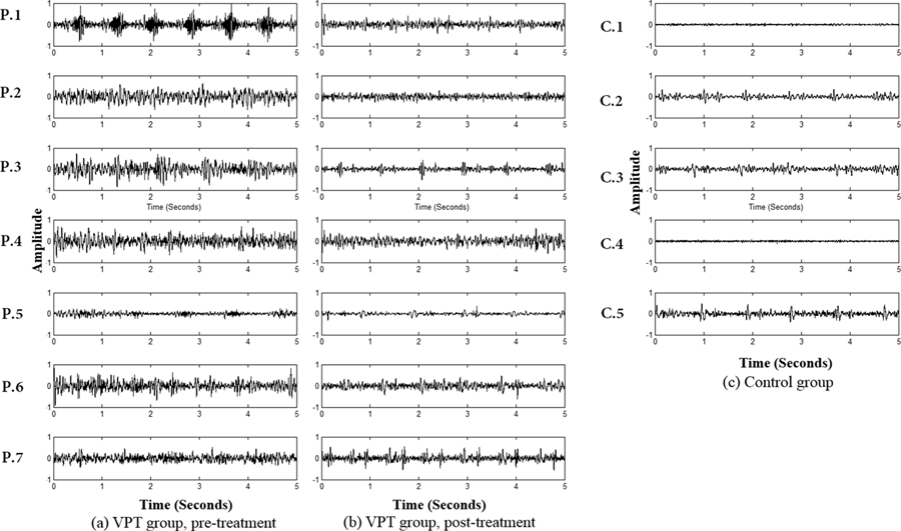
Pre-treatment (A) and post-treatment (B) ear canal signals of seven vascular pulsatile tinnitus subjects and ear canal signals of five control subjects (C) measured with an upright, neutral head position.

**Fig 2:**
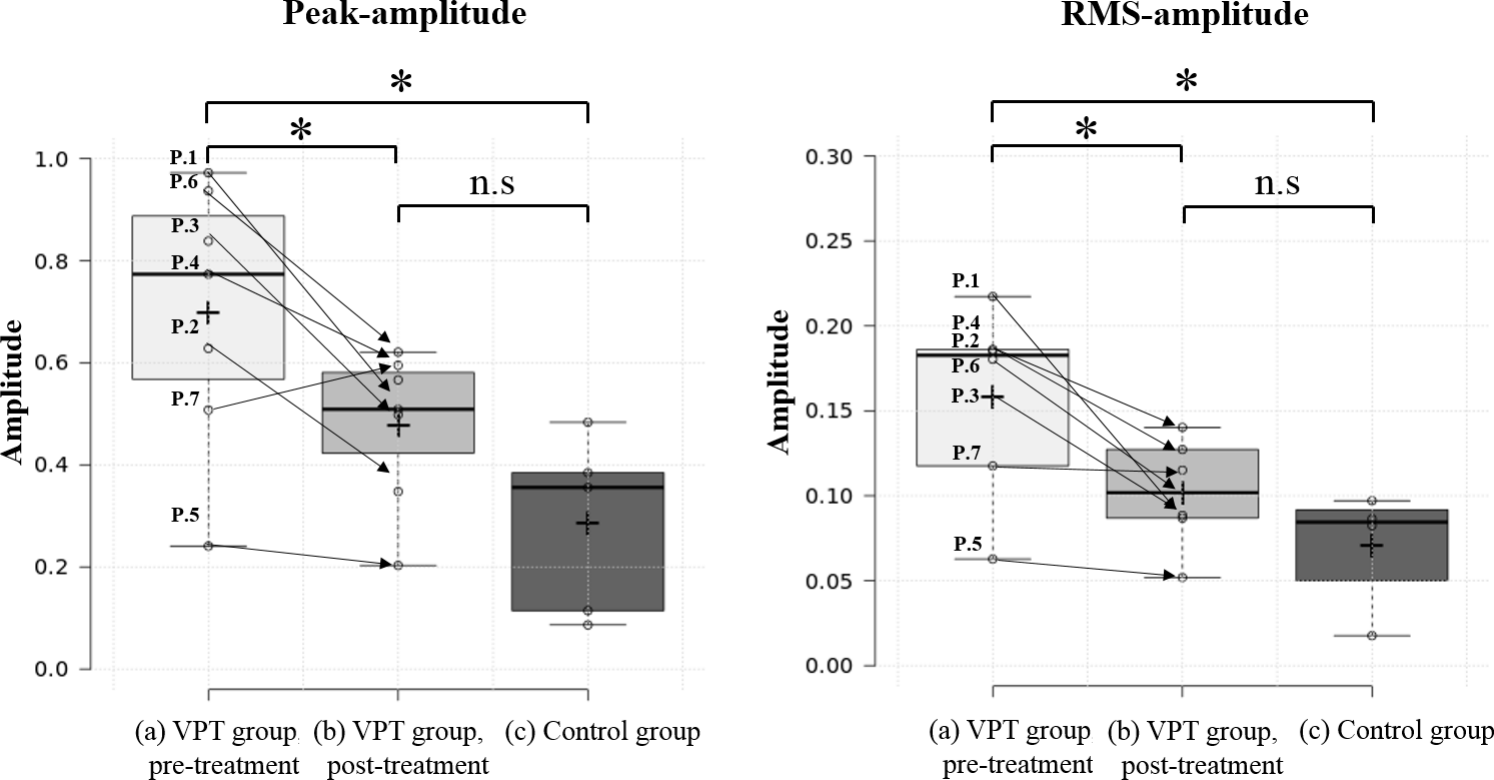
**Differences between pre- and post-treatment ear canal signals of the patient group and ear canal signals of the control group analyzed in the time domain with regard to peak-amplitudes (A) and root mean square-amplitudes (B).** Asterisks designate statistically significant differences. n.s., non-significant.

Of the signals recorded in eight different head positions, the signals acquired from the upright, neutral head position were selected and analyzed for signal comparison. As illustrated in Fig 1, the pre- and post-treatment ear canal signals (S1–S14 Files) (the left and right columns, respectively) from seven VPT patients and the ear canal signals of the five controls (S15–S19 Files) measured in the upright position with a neutral head were analyzed with time domain. While the pre-treatment signals exhibited large peak amplitudes and periodic structures, the post-treatment signals had weaker waveforms than the pre-treatment signals in all seven subjects. Meanwhile, the signals of the control subjects had smaller peak amplitudes and less periodicity. As shown in Fig 1, reduced peak amplitudes and RMS amplitudes were observed among all patients after surgical treatment. Compared with the peak-amplitudes of the pre-treatment signals (median, 0.77; range, 0.24–0.97), those of the post-treatment signals (median, 0.52; range, 0.2–0.62) showed a significant decrease (Fig 2A, *P* < 0.05, Wilcoxon signed-rank sum test). The RMS amplitude of the pre-treatment signals (median, 0.18; range, 0.06–0.22) also showed a significant decrease after treatment (median, 0.11; range, 0.05–0.14) (Fig 2B, *P* < 0.05, Wilcoxon signed-rank sum test). While the pre-treatment peak and RMS amplitudes of the VPT subjects were significantly larger than those of the controls, there were no significant differences between the signals measured after treatment in the VPT group and those of the control group with regard to both peak and RMS amplitudes.

### Spectro-temporal analysis (time-frequency domain analysis)

**Fig 3:**
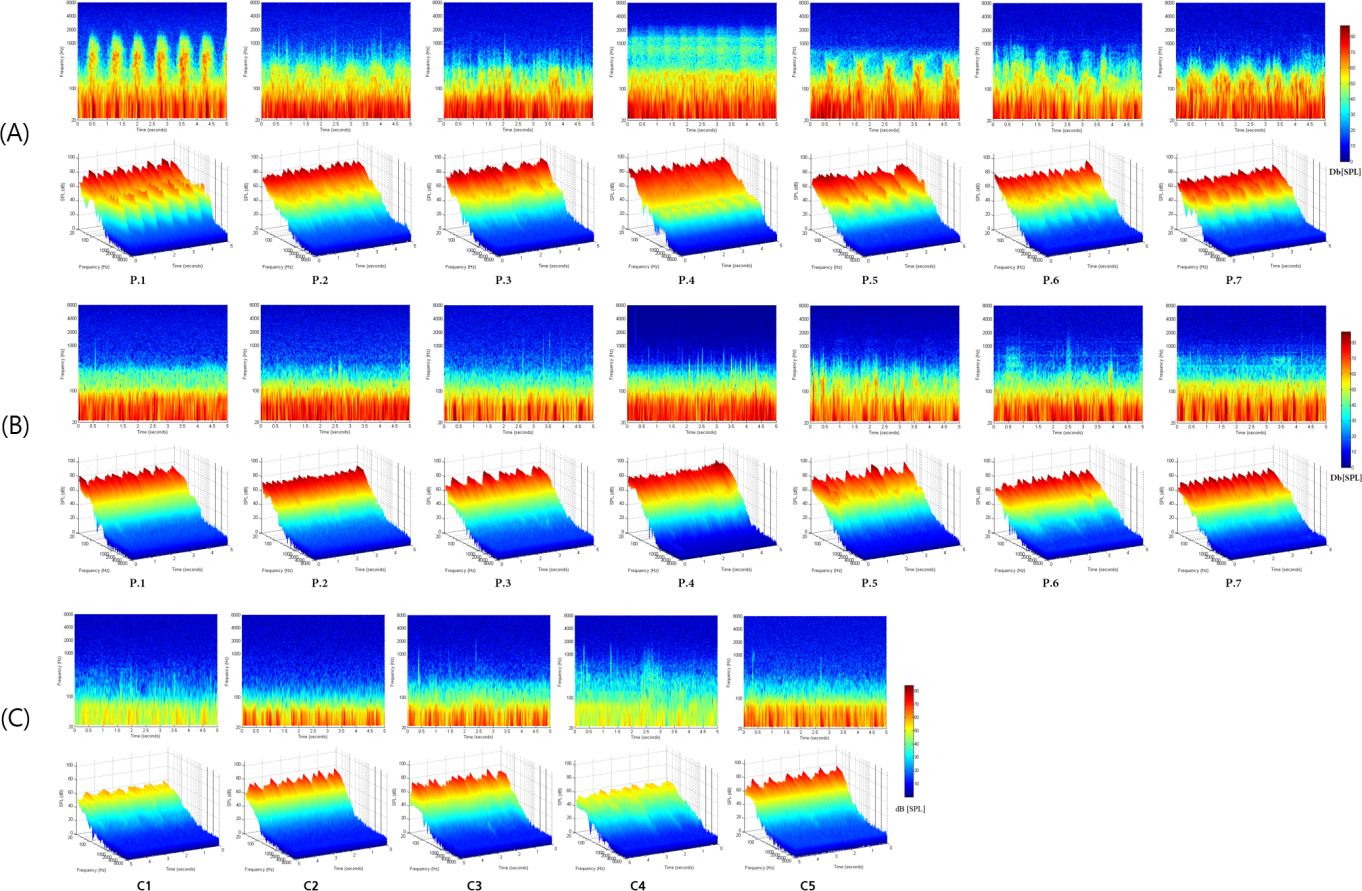
Two-dimensional and three-dimensional spectrogram of the recorded pre-treatment (A) and post-treatment (B) ear canal signals of the patient group and that of the control group (C).

STA of the recorded signals provided further observable information regarding each subject’s VPT and its improvement after treatment, in addition to that gained from the time domain analysis. The 2D and 3D spectrograms of the pre- and post-treatment signals are shown in Fig 3. All seven VPT subjects exhibited spectro-temporal characteristic ear canal signals that were well in line with their presumptive origins (Fig 3A). For example, the 2D and 3D spectrograms of the recorded signal in Subjects 1, 5 (sigmoid sinus diverticulum) and Subject 7 (dominant transverse-sigmoid sinus with bony dehiscence) revealed the pulse-synchronous broadband nature of audible SPLs, whereas Subjects 2, 6 (dehiscent jugular bulb) and Subject 3 (dominant transverse-sigmoid sinus with high jugular bulb) exhibited pulse-synchronous audible SPLs primarily at a relatively low-frequency range. Subject 4 (dural arteriovenous fistula) exhibited peculiar pulsatile bumps at approximately 1,000 Hz, which were not observed in the other 4 patients.

**Fig 4:**
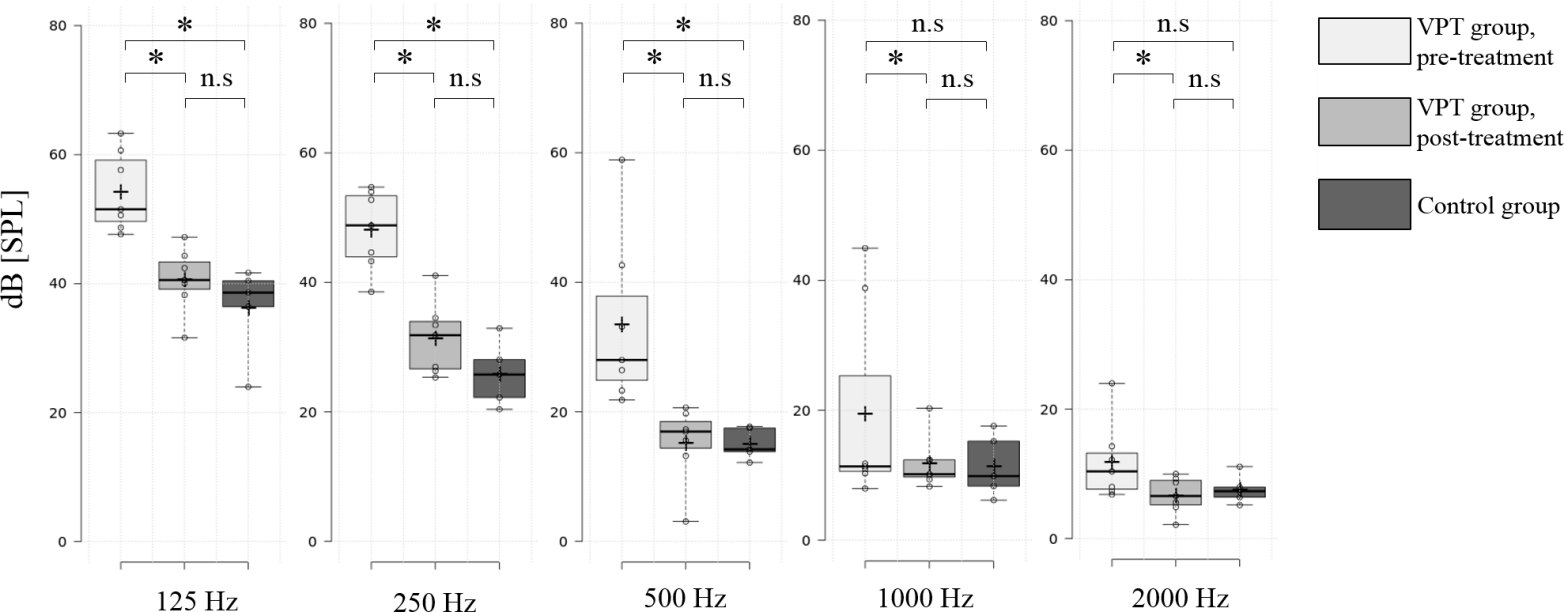
Sound pressure level differences of the pulse-synchronized five spectral bands (~50ms) between pre- and post-treatment ear canal signals of the patient group and ear canal signals of the control group analyzed in the frequency domain. Asterisks designate statistically significant. n.s., non-significant.

Following surgical treatment, all subjects displayed no or much decreased pulse-synchronous signal in their spectrograms and, in particular, the low-frequency components of the signals were much decreased compared with the pre-treatment signals (Fig 3B). This change was further validated by comparing the SPL differences of the five pulse-synchronized spectral bands (125, 250, 500, 1,000, and 2,000 Hz). As shown in Fig 4, SPL changes were found to be statistically significant at all frequencies (*P* < 0.05, Wilcoxon signed-rank sum test), but the differences were starker at low frequencies (125, 250, and 500 Hz) as compared with those at 1,000 and 2,000 Hz. Compared with the control group, as shown in Fig 4, the VPT patients showed significantly larger spectral bands at 125, 250, and 500 Hz before treatment (*P* < 0.05, Mann Whitney U-test), but the post-treatment spectral bands showed no significant differences compared to the control group. In other words, postoperatively, the patients were not different from the controls with regard to pulse-synchronized spectral bands.

## Discussion

As mentioned previously, approximately 80% of VPT cases are not objectively detected by a conventional physical examination. With this in mind, we recently assessed five subjective VPT participants with presumptive vascular origins by applying the TSR/STA method and extracted objective acoustic features that were representative of the underlying vascular pathology [16]. In other words, the TSR/STA method was able to detect “hidden objective VPT” that would otherwise be inaudible by bare-ear auscultation.

In the current study, we further evaluated the feasibility of the refined TSR/STA method, not only for pre-treatment objective diagnosis, but also for the post-treatment evaluation of the changes in seven VPT patients who were treated by surgical- or endovascular intervention. Namely, we compared the pre- and post-treatment ear canal signals to determine the differences in signal with regard to time and frequency domains, and also compared the pre- and post-treatment signals to the signals of normal controls without VPT. As stated above, reduced peak amplitudes and RMS amplitudes with statistical significance were observed using a time domain analysis among all patients after surgical treatment. In addition, in time-frequency domain analysis, the pulse-synchronous signal of all subjects was not identifiable and the SPL decrement was statistically significant at low frequencies, such as 125, 250, and 500 Hz. Moreover, while the pre-treatment peak and RMS amplitudes, as well as the SPL at low frequency spectral bands, were significantly larger than those of the controls, no significant differences were found between the post-treatment signals and the control group’s signal with regard to the same parameters.

### Replicated identification of “hidden objective pulsatile tinnitus” by the refined TSR/STA method

In the current case series, all seven subjects had “subjective” VPT that could not be confirmed by bare-ear auscultation. However, through the use of our refined portable transcanal recording system, which solves the ground loop problem, it was possible to obtain characteristic and documentable signals representative of the presumptive vascular pathologies responsible for VPT perception. That is, all seven VPT subjects exhibited pulse-synchronous ear canal signals (Figs 1 and 3) that were not observed in the control subjects, and the signals exhibited disease-specific characteristics.

For example, Subjects 1, 5 (sigmoid sinus diverticulum) and Subject 7 (dominant sigmoid sinus with bony dehiscence) showed a relatively large rise and fall compared with the other four subjects, and this may indicate a distinctly turbulent flow generated in the sigmoid sinus and transmitted to the middle ear through the bony dehiscence. In contrast, Subjects 2 and 3 showed pulse-synchronous signals, but these were less obvious than in Subjects 1, 5, and 7. This may be attributed to their pathologies (Subject 2, dehiscent jugular bulb; Subject 3, dominant transverse-sigmoid sinus with high jugular bulb) in that they may transmit venous blood flow to the middle ear but without any stark turbulence.

The ear canal signal of Subject 4 had a unique bump-like rise and fall at approximately 1,000 Hz, which was also observed in the subject with dural arteriovenous fistula in our previous study. This may reflect sound conduction from fast blood flow in the fistulous tract to the middle ear. In summary, when distinct pathology exists in or near the middle ear, we can obtain disease-specific ear canal signals using the refined TSR/STA method in selected cases. These results may be of value in that they were able to replicate the results of our previous study with regard to signal detection and discrete characteristics according to pathology.

### Objective evaluation of treatment results by comparing pre- and post-treatment signals

In selected VPT cases with an obvious pathology, treatments such as surgical repair of the vascular pathology or endovascular embolization are applicable for the improvement of symptoms [13,14,18,19]. However, the selection of a proper treatment modality or the method of evaluating treatment results is limited because most VPTs are only subjectively perceived. Therefore, the evaluation of results of treatment from previous studies has been based on subjectively-perceived symptom changes or changes in tinnitus questionnaire scores using subjective answers.

Using the current methodology, we were able to objectively demonstrate post-treatment changes with regard to the spectral and temporal characteristics of the recorded signal. In time domain analysis, both peak amplitudes and RMS amplitudes significantly decreased in all subjects after surgical or interventional treatment. In addition, in time-frequency domain analysis, the pulse-synchronous signal of all subjects had abated and, in particular, the SPL significantly decreased at low frequencies. These changes in SPL at low frequency components may explain why most VPT patients perceive a low frequency humming sound and why they do not perceive such pulsatile sounds after proper intervention. Moreover, by comparing the pre- and post-treatment data to that of the control group, we could objectively explain why patients no longer heard their VPT after proper treatment. In other words, after proper treatment, the VPT subjects did not differ from the controls, with no history of VPT perception, with regard to the peak and RMS amplitudes, as well as the SPL, at low frequency spectral bands.

These results confirmed, in an objective way, the successful control of VPT after treatment and were used for post-treatment patient consultation. By comparing the pre- and post-treatment signals objectively, clinicians can confirm the effectiveness of treatment and reassure patients with regard to the surgical outcome by visualizing the signal changes. The TSR/STA method may be of great value because it not only detects hidden objective VPT, but also objectively demonstrates post-treatment improvement and, therefore, the future management of similar vascular pathologies can be based on an objective treatment outcome.

### Limitations of the current study and future directions

Although the current study demonstrated the potential of the TSR/STA method in evaluating treatment outcomes, several limitations should be addressed. First, in order to verify this method, future studies with a larger number of participants should be conducted to better characterize the role of the TSR/STA method. By accumulating a larger data set from future subjects with diverse etiologies, we may be able to further characterize and objectify presumed vascular pathologies and the expected treatment outcome. In addition, there are likely to be cases of VPT in which TSR shows no demonstrable signals, and in such cases the TSR/STA method may aid in choosing proper diagnostic imaging modalities, counseling the patients, and deciding proper therapeutic options. Second, the current method requires further refinement with regard to the insertion depth of the microphone and the inter-individual morphological variations in the external and middle ear. Although we used in-ear foam tips attached at the end of the microphone to fit the microphone maximally to the ear canal, the prototype recording system may become more objective and precise by individualizing the size of the inserted microphone or utilizing tympanic membrane contact-type microphones.

## Conclusions

Taken together, we further demonstrated that the TSR/STA method is an effective modality to objectify VPT. Additionally, the potential role of the TSR/STA method in the objective evaluation of treatment outcomes in patients with VPT was shown by comparing the pre- and post-treatment ear canal signals. For routine use of the current method in evaluating patients with VPT, future studies with a larger sample size and more refined recording techniques are warranted.

## Supporting Information

S1 File Audio file of pre-treatment ear canal signal from the no.1 VPT patient, shown in Fig 1A. (WAV)

S2 File Audio file of post-treatment ear canal signal from the no.1 VPT patient, shown in Fig 1B. (WAV)

S3 File Audio file of pre-treatment ear canal signal from the no.2 VPT patient, shown in Fig 1A. (WAV)

S4 File Audio file of post-treatment ear canal signal from the no.2 VPT patient, shown in Fig 1B. (WAV)

S5 File Audio file of pre-treatment ear canal signal from the no.3 VPT patient, shown in Fig 1A. (WAV)

S6 File Audio file of post-treatment ear canal signal from the no.3 VPT patient, shown in Fig 1B. (WAV)

S7 File Audio file of pre-treatment ear canal signal from the no.4 VPT patient, shown in Fig 1A. (WAV)

S8 File Audio file of post-treatment ear canal signal from the no.4 VPT patient, shown in Fig 1B. (WAV)

S9 File Audio file of pre-treatment ear canal signal from the no.5 VPT patient, shown in Fig 1A. (WAV)

S10 File Audio file of post-treatment ear canal signal from the no.5 VPT patient, shown in Fig 1B. (WAV)

S11 File Audio file of pre-treatment ear canal signal from the no.6 VPT patient, shown in Fig 1A. (WAV)

S12 File Audio file of post-treatment ear canal signal from the no.6 VPT patient, shown in Fig 1B. (WAV)

S13 File Audio file of pre-treatment ear canal signal from the no.7 VPT patient, shown in Fig 1A. (WAV)

S14 File Audio file of post-treatment ear canal signal from the no.7 VPT patient, shown in Fig 1B. (WAV)

S15 File Audio file of ear canal signals from the no.1 control, shown in Fig 1C. (WAV)

S16 File Audio file of ear canal signals from the no.2 control, shown in Fig 1C. (WAV)

S17 File Audio file of ear canal signals from the no.3 control, shown in Fig 1C. (WAV)

S18 File Audio file of ear canal signals from the no.4 control, shown in Fig 1C. (WAV)

S19 File Audio file of ear canal signals from the no.5 control, shown in Fig 1C. (WAV)
